# Prevalence and determinants of hypertension among people living with HIV in Ghana: A cross-sectional study

**DOI:** 10.1371/journal.pone.0342198

**Published:** 2026-02-12

**Authors:** Kasim Abdulai, Safianu Osman Aleboko, Isaac Anane, Ivan Addae-Mensah, Abdul-Malik Bawah

**Affiliations:** 1 Translational Nutrition Research Group, Department of Nutrition and Dietetics, University of Cape Coast, Cape Coast, Ghana; 2 Department of Nutritional Sciences, Oklahoma State University, Stillwater, Oklahoma, United States of America; 3 Department of Nutrition and Dietetics, Faculty of Allied Health and Pharmaceutical Sciences, Tamale Technical University, Ghana; Fayetteville State University, UNITED STATES OF AMERICA

## Abstract

**Background:**

Hypertension is a major health concern in Sub-Saharan Africa (SSA). People living with human immunodeficiency virus (PLHIV) face unique risks for cardiometabolic disorders. However, the factors associated with hypertension among PLHIV have been understudied in SSA. This study examines the prevalence and determinants of hypertension among PLHIV in Ghana.

**Methods:**

A total of 440 PLHIV aged 18 years and older receiving antiretroviral therapy (ART) for a minimum of six months were recruited in this hospital-based cross-sectional study. Variables assessed included blood pressure, alcohol consumption, type of antiretroviral therapy (ART) medication, duration of exposure to ART, smoking history, age, sex, level of education, and exercise. Binary logistic regression was used to determine the factors associated with hypertension.

**Results:**

The overall prevalence of hypertension was 33%. People living with HIV who were on second-line ART had a lower risk of hypertension compared to those on first-line ART (OR = 0.379; 95% CI: 0.169–0.846; p = 0.018). Similarly, participants with high muscle mass (OR = 0.177; 95% CI: 0.064–0.487; p < 0.01) and those with very high muscle mass (OR = 0.220; 95% CI: 0.051–0.943; p = 0.041) had a lower risk of hypertension compared to those with low muscle mass. In contrast, participants who were obese had approximately four times greater odds of hypertension compared to those with underweight (OR = 4.046; 95% CI: 1.018–16.083; p = 0.047). Additionally, participants with medium IDDS (OR = 1.968; 95% CI: 1.150–3.369; p = 0.014) and high IDDS (OR = 2.348; 95% CI: 1.078–5.115; p = 0.032) had about twice the risk of hypertension compared to those with low IDDS.

**Conclusion:**

This study found a high prevalence of hypertension among PLHIV. Second-line ART may reduce the risk of hypertension, while higher muscle mass may have a protective effect. Further research is needed to better understand the impact of dietary diversity and specific dietary components on hypertension in this population.

## Introduction

Hypertension remains a serious global public health issue that greatly increases the burden of kidney failure, stroke, and cardiovascular illnesses. In Sub-Saharan Africa (SSA), including Ghana, the prevalence of hypertension has been rising steadily over the past few decades, driven by rapid urbanization, lifestyle changes, and the increasing prevalence of noncommunicable diseases (NCDs) [1–3]. Within this context, PLHIV represent a particularly vulnerable group, as they face unique challenges that may predispose them to a higher risk of developing hypertension [4–7]. According to the Ghana AIDS Commission’s 2024 National and Sub-National HIV and AIDS Estimates and Projections Report, an estimated 334,721 people are living with HIV in Ghana, comprising 316,492 adults (94.6%) and 18,229 children under 15 years (5.4%). Regionally, the Greater Accra Region recorded the highest number of PLHIV (77,821; 23.2%), followed by the Ashanti Region (63,159; 18.9%) and the Eastern Region (44,792; 13.4%). The North East Region recorded the lowest PLHIV population (1,717; 0.5%) [8].

Ghana, a lower-middle-income country in West Africa, has made significant strides in the fight against HIV/AIDS, with substantial investments in ART and other health infrastructure [8]. As a result, the life expectancy of PLHIV has increased, transforming HIV from a fatal disease to a chronic condition that can be managed with appropriate treatment [8–10]. However, this success has also led to new health challenges, including the rising prevalence of NCDs, such as hypertension, among PLHIV. The intersection of HIV and hypertension is a growing concern in Ghana, as both conditions can exacerbate each other and complicate disease management, leading to increased morbidity and mortality [9]. Understanding the prevalence and factors associated with hypertension among PLHIV in Ghana is critical for informing effective public health interventions aimed at improving the health and well-being of this population.

Several factors contribute to the heightened risk of hypertension among PLHIV in Ghana. First, the use of ART, particularly certain classes of drugs such as protease inhibitors, has been associated with metabolic changes, including dyslipidemia, insulin resistance, and fat redistribution, all of which can increase the risk of hypertension [11,12]. Additionally, the chronic inflammation and immune activation associated with HIV infection itself may contribute to vascular damage and elevated blood pressure [13,14]. Furthermore, lifestyle factors common among the general Ghanaian population, such as high salt intake, physical inactivity, and excessive alcohol consumption, may also play a role in the development of hypertension among PLHIV [15].

The social determinants of health, including socioeconomic status, education level, and access to healthcare, are also important considerations in understanding the prevalence of hypertension among PLHIV in Ghana [16,17]. Many PLHIV in Ghana face significant barriers to accessing healthcare, including stigma and discrimination, which may prevent them from seeking regular medical care and monitoring their blood pressure [16,18]. Moreover, the high cost of healthcare services and medications can be prohibitive for many Ghanaians, particularly those living in rural areas or with limited financial resources [19].

The prevalence of hypertension among PLHIV in Ghana is likely to be influenced by a combination of these biological, behavioral, and social factors [20,21]. Despite the growing recognition of the possible link between HIV and hypertension, there is a paucity of data on the prevalence and determinants of hypertension among PLHIV in Ghana. This gap in knowledge underscores the need for targeted research to elucidate the extent of the problem and identify modifiable risk factors that can be addressed through public health interventions. This study therefore aimed to determine the prevalence and determinants of hypertension among people living with HIV receiving ART in the Lower Manya Krobo Municipality, Ghana.

## Methods

### Study design and settings

A hospital-based cross-sectional study was conducted at two hospitals in the Lower Manya Krobo Municipality of the Eastern Region of Ghana. The municipality is in the south-eastern part of Ghana, approximately 85 kilometers from Accra, the national capital. According to the 2021 Population and Housing Census, the Eastern Region has an estimated population of about 2.9 million, while the Lower Manya Krobo Municipality accounts for approximately 121,000 inhabitants. The municipality is largely peri-urban, with a youthful population structure, a high literacy rate relative to the national average, and a predominance of the Krobo ethnic group, although other ethnicities such as Ewe, Akan, and Ga-Adangbe are also represented. Most residents engage in petty trading, farming, or artisanal work. The Eastern Region ranks among the top three regions with the highest HIV prevalence in Ghana. Hypertension, diabetes, and other non-communicable diseases are also recognized as emerging health challenges, although population-based prevalence data are limited.

The study was carried out in two hospitals providing comprehensive HIV care services. The first, Atua Government Hospital, is a state-owned secondary-level facility located in Odumase Krobo, serving as the principal referral center for the municipality and neighboring districts. The hospital provides ART, antenatal, outpatient, and inpatient services and has a catchment population of approximately 200,000. The second facility, St. Martin de Porres Hospital, is a faith-based (Catholic) hospital situated in Agormanya, also in the Lower Manya Krobo Municipality. It operates as a general hospital providing medical, surgical, maternal, and child health services. Both hospitals run dedicated ART clinics established under the National AIDS/STI Control Programme and have fully functional hypertension and diabetes clinics. Laboratory services include facilities for CD4 count, viral load testing, hematology, and basic biochemistry. Each hospital has a multidisciplinary team comprising physicians, nurses, counselors, pharmacists, and laboratory scientists trained in ART management and chronic disease care. Both hospitals were selected for the study because they provide ART services, which makes them suitable settings for investigating the study’s objectives. The inclusion criteria required participants to be PLHIV who were 18 years or older, had been on ART for at least six months, and were able to provide informed written consent. Pregnant and lactating women living with HIV, individuals on special diets, and PLHIV who visited the hospital for reasons other than ART were excluded. The research was carried out between February 2020 and June 2020.

### Sampling procedure

This study’s sample size was calculated using Cochran’s formula for estimating a single population proportion, with hypertension prevalence among PLHIV as the primary outcome variable [22]. The computation assumed a 95% confidence level (z = 1.96), a presumed prevalence (p) of 0.50: selected to maximize variability in the absence of reliable local estimates, and a margin of error (d) of 0.05. To account for a potential 15% non-response rate, reflecting expected losses due to incomplete questionnaires, or ineligible records, the final target sample size was 440 participants. This conservative estimation was to provide sufficient statistical power analyses and multivariable modeling. Participants were allocated between two study sites, 228 at Atua Government Hospital and 212 at St. Martin De Porres Hospital, using Probability Proportional to Size (PPS) based on the number of active ART clients registered at each facility. During routine ART clinic sessions, which typically recorded an average attendance of approximately 50 clients at Atua Government Hospital and 60 at St. Martin De Porres, 20 and 25 participants, respectively, were consecutively recruited per day until the target numbers were achieved.

### Measures

Data were collected between February and June 2020 using a structured interviewer-administered questionnaire and standardized clinical measurement tools. Research assistants captured responses using mobile phones equipped with the Open Data Kit (ODK) Collect App. Prior to fieldwork, all assistants received extensive training and were assessed for competence in administering the questionnaire, conducting measurements, and using equipment according to standardized protocols. The questionnaire and digital tool were pretested with 15 adults in a comparable population. All data were reviewed daily for accuracy and completeness by the field supervisors and verified by the principal investigator.

### Blood pressure assessment

Blood pressure, the primary outcome of interest, was assessed using a calibrated automatic digital sphygmomanometer (Omron M3). Participants were seated comfortably with their dominant arm supported at heart level and were asked to rest for a minimum of five minutes in a quiet setting prior to measurement. Three readings were taken at two-minute intervals, and the average of the three readings was used to determine final blood pressure status. Hypertension was defined as systolic blood pressure ≥140 mmHg and/or diastolic blood pressure ≥90 mmHg, following internationally recognized clinical thresholds. This measurement protocol adheres to World Health Organization (WHO) standards and has been validated for epidemiological research.

### Anthropometry and body composition

Anthropometric and body composition measurements were taken to assess participants’ nutritional and metabolic status. Body composition was evaluated using the Omron Body Composition Monitor and Scale (Model HBF-516) that estimates body fat and lean muscle mass by measuring resistance to a small electrical current. The device was calibrated daily, and participants were instructed to remove any metal items and stand barefoot and still on the machine’s footplates during measurement. Additional anthropometric data were obtained by measuring height with a portable stadiometer and weight with a calibrated digital scale. Body mass index (BMI) was calculated as weight (kg) divided by height squared (m^2^) and categorized using WHO classifications: underweight (<18.5), normal (18.5–24.9), overweight (25.0–29.9), and obese (≥30.0).

### Antiretroviral therapy and clinical information

Information on participants’ ART was extracted from clinical records after obtaining informed consent. This included ART regimen type (first-line or second-line) and duration on therapy (in months). These variables were included based on their potential role in modifying cardiovascular and metabolic risk among people living with HIV. Data extraction was guided by a predesigned abstraction form to ensure consistency and accuracy across the two study sites.

### Lifestyle and sociodemographic characteristics

Participants provided information on lifestyle behaviors including alcohol use, smoking status, and physical activity through structured close-ended questions adapted from national health surveys. Alcohol and smoking variables were captured as binary (yes/no), while physical activity was assessed based on frequency and intensity, and categorized accordingly. Sociodemographic characteristics such as age, sex, and level of education were recorded.

### Dietary diversity assessment

Dietary data were collected using a structured 24-hour dietary recall questionnaire. The questionnaire was adopted from the Food and Agriculture Organization (FAO) guidelines for measuring household and individual dietary diversity [23]. The 24-hour dietary recall questionnaire administered without modification, as it has been validated for use in low-resource and population-based nutrition studies. Participants were asked to recall all foods and beverages consumed in the preceding 24 hours, which were subsequently classified into 12 standard FAO food groups: cereals; roots and tubers; legumes and nuts; dairy products; meat, poultry, and fish; eggs; dark green leafy vegetables; vitamin A–rich fruits and vegetables; other vegetables; other fruits; oils and fats; and sweets. Responses from the 24-hour recall questionnaire were used to compute the Individual Dietary Diversity Score (IDDS). Each food group consumed was assigned a score of 1, and food groups not consumed were assigned a score of 0, resulting in a total IDDS ranging from 0 to 12. Higher scores indicate greater dietary diversity and are considered a proxy measure of micronutrient adequacy. All interviews were conducted by trained research assistants using structured interviews, and responses were recorded electronically using the ODK platform to ensure standardized data capture and minimize transcription errors.

### Data analysis

All statistical analyses were conducted using IBM SPSS Statistics (version 20) [24]. Descriptive statistics including means, standard deviations, frequencies, and percentages were used to summarize sociodemographic characteristics and key study variables such as BMI, muscle mass, ART regimen, and hypertension status. Chi-square tests were conducted to explore associations between hypertension and categorical variables such as education level, occupation, ART type, dietary diversity, and body composition indicators. Where necessary, Fisher’s exact test was applied for cells with small expected counts.

Binary logistic regression was employed to identify factors independently associated with hypertension among PLHIV. Variables included in the regression model were selected based on theoretical relevance and significant bivariate associations. Predictor variables included ART regimen (first-line vs. second-line), duration on ART, BMI category, muscle mass, body fat percentage, IDDS, smoking status, alcohol consumption, and key sociodemographic variables. Reference categories were designated for each categorical variable to allow for clear interpretation of odds ratios (ORs). Multicollinearity was assessed a priori using variance inflation factors (VIFs) to ensure the independence of predictor variables. Effect estimates were reported as odds ratios with corresponding 95% confidence intervals (CIs) and p-values. The model’s overall fit was evaluated using the Chi-square test of model significance and the Nagelkerke pseudo-R^2^ statistic to estimate the proportion of variance explained. Statistical significance was set at p < 0.05. All tests were two-sided.

### Ethical considerations

The study received ethical approval from the Ghana Health Service Ethics Review Committee (GHS-ERC) under protocol number GHS-ERC 007/07/19. Informed consent was obtained from participants through signed consent forms, and for individuals unable to read or write, the documents were read and explained to them, with consent recorded using a thumbprint. Confidentiality and privacy of participant data were prioritized throughout the research process, with digital data stored securely through encrypted access and physical forms kept in locked, secure locations to ensure data security.

## Results

### Sociodemographic characteristics of study participants

Table 1 presents the socio-demographic characteristics of the 440 participants. The mean age of participants was 49.28 years (SD = ±11.96). The study population was predominantly female (85%). Participants were recruited from two facilities, with 51.9% attending Atua and 48.1% from St. Martins. The majority of participants (56.7%) were aged 35–54 years. Education levels showed that 45.1% had a middle/secondary education, while 25.2% had no formal education. The most common occupation was trading (57.8%), with the majority of men being farmers (37.3%) and artisans (35.8%), while most women were traders (66.8%).

**Table 1:** Socio-demographic characteristics of participants.

Characteristics	Female, n (%) *372 (85)	Male, n (%) **67 (15)	Total, n (%) 440 (100)
**Facility**			
Atua	197 (52.7)	32 (47.8)	229 (51.9)
St. Martins	177 (47.3)	35 (52.2)	212 (48.1)
**Age Group**			
18–34 years	43 (11.5)	7 (11.5)	50 (11.3)
35–54 years	225 (60.2)	25 (37.3)	250 (56.7)
55 + years	106 (28.3)	35 (52.2)	141 (32.0)
**Level of Education**			
None	108 (28.9)	3 (4.5)	111 (25.2)
Primary	73 (19.5)	12 (17.9)	85 (19.3)
Middle/Secondary	159 (42.5)	40 (59.7)	199 (45.1)
Higher	9 (2.4)	4 (6.0)	13 (2.9)
**Marital Status**			
Single	114 (30.6)	14 (20.9)	128 (29.1)
Married/Cohabiting	100 (26.9)	37 (58.3)	137 (31.1)
Divorced/Separated	43 (11.6)	5 (7.5)	48 (10.9)
Widowed	116 (31.1)	11 (16.4)	127 (28.9)
**Occupation**			
Unemployed	44 (11.8)	5 (7.5)	49 (11.1)
Farmer	14 (3.7)	25 (37.3)	39 (8.8)
Artisan	41 (11.0)	24 (35.8)	65 (14.7)
Formal Public Sector	19 (5.1)	2 (3.0)	21 (4.8)
Formal Private Sector	5 (1.3)	4 (6.0)	9 (2.0)
Trading	250 (66.8)	5 (7.5)	255 (57.8)
Others	1 (0.3)	2 (3.0)	3 (0.7)
**Ethnicity**			
Akan	22 (5.9)	4 (6.0)	26 (5.9)
Ga/Dangme	302 (80.7)	57 (85.1)	359 (81.4)
Ewe	40 (10.7)	4 (6.0)	44 (10.0)
Others	10 (2.7)	2 (3.0)	12 (2.7)
**Religion**			
Christianity	367 (98.1)	66 (98.5)	433 (98.2)
Islam	5 (1.3)	1 (1.5)	6 (1.4)
Traditional	2 (0.5)	0 (0.0)	2 (0.5)

*Total number of females (n = 373).

**Total number of males (n = 67).

The mean age of the participants was 49.28 years (SD = ±11.96).

### Prevalence of hypertension

**Fig 1:**
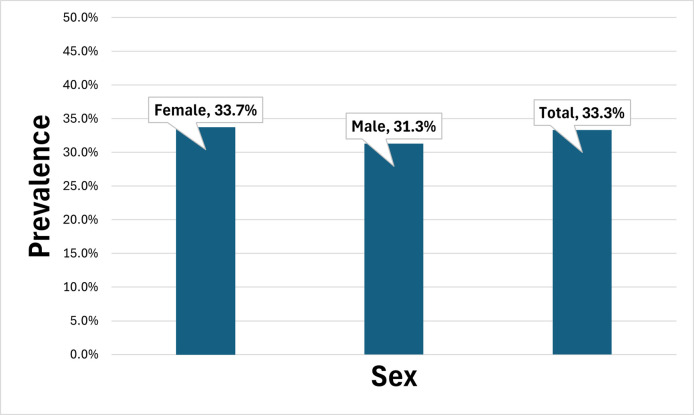
Prevalence of hypertension.

The prevalence of hypertension among the PLHIV was also examined. The results show hypertension prevalence was higher among females (33.7%) in the study, as against prevalence of 31.3% among males (Fig 1). The overall prevalence of hypertension among PLHIV was about 33.3%.

Table 2 presents the association between various explanatory factors and hypertension among participants. Significant associations were identified for marital status (χ² = 11.763, p = 0.038), occupation (χ² = 18.865, p = 0.004), ART regimen type (χ² = 4.889, p = 0.027), IDDS category (χ² = 7.907, p = 0.019), BMI category (χ² = 11.762, p = 0.008), body fat category (χ² = 12.537, p = 0.006), and muscle mass category (χ² = 18.595, p= < 0.001). Factors such as level of education, age category, smoking status, alcohol consumption, duration of medication, and visceral fat category did not show statistically significant associations with hypertension (p > 0.05).

**Table 2:** Chi-square table for explanatory variables and hypertension.

Factor	BP Status		Total, n (%)	Chi Square	p-value
	Normal, n (%)	Hypertensive, n (%)			
**Level of Education**					
None	70 (63.1)	41 (36.9)	111 (100.0)	2.413	.660
Primary	56 (65.9)	29 (34.1)	85 (100.0)		
Middle/JHS	138 (69.3)	61 (30.7)	199 (100.0)		
Secondary/SHS	23 (69.7)	10 (30.3)	33 (100.0)		
Higher	7 (53.8)	6 (46.2)	13 (100.0)		
**Age category**					
18–34 years	100 (75.2)	33 (24.8)	133 (100.0)	5.542	.063
35–54 years	155 (63.8)	88 (36.2)	243 (100.0)		
55 + years	38 (63.3)	22 (36.7)	60 (100.0)		
**Marital Status**					
Single	91 (71.1)	37 (28.9)	128 (100.0)	11.763	.038**
Married	52 (56.5)	40 (43.5)	92 (100.0)		
Divorced	26 (76.5)	8 (23.5)	34 (100.0)		
Widowed	79 (62.2)	48 (37.8)	127 (100.0)		
Separated	10 (71.4)	4 (28.6)	14 (100.0)		
Cohabiting	36 (80.0)	9 (20.0)	45 (100.0)		
**Occupation**					
Unemployed	33 (67.3)	16 (32.7)	49 (100.0)	18.865	.004*
Farmer	32 (82.1)	7 (17.9)	39 (100.0)		
Artisan	53 (81.5)	12 (18.5)	65 (100.0)		
Public Sector	15 (71.4)	6 (28.6)	21 (100.0)		
Private Sector	4 (44.4)	5 (55.6)	9 (100.0)		
Trading	154 (60.4)	101 (39.6)	255 (100.0)		
**Ever Smoked**					
No	268 (65.7)	140 (34.3)	408 (100.0)	2.358	.125
Yes	26 (78.8)	7 (21.2)	33 (100.0)		
**Ever consumed Alcohol**					
No	67 (67.7)	32 (32.3)	99 (100.0)	.059	.809
Yes	227 (66.4)	115 (33.6)	342 (100.0)		
**Type of ART**					
First line ART	253 (64.9)	137 (35.1)	390 (100.0)	4.889	.027**
Second line ART	41 (80.4)	10 (19.6)	51 (100.0)		
**Duration of medication**					
< 6 months	20 (60.6)	13 (39.4)	33 (100.0)	.734	.865
6 months - < 12 months	18 (66.7)	9 (33.3)	27 (100.0)		
1- < 4 years	63 (65.6)	33 (34.4)	96 (100.0)		
4 years and above	193 (67.7)	92 (32.3)	285 (100.0)		
**IDDS category**					
Low	41 (82.0)	9 (18.0)	50 (100.0)	7.907	.019*
Medium	168 (67.2)	82 (32.8)	250 (100.0)		
Higher	85 (60.3)	56 (39.7)	141 (100.0)		
**BMI Category**					
Underweight	49 (76.6)	15 (23.4)	64 (100.0)	11.762	.008*
Normal	158 (70.2)	67 (29.8)	225 (100.0)		
Overweight	65 (59.6)	44 (40.4)	109 (100.0)		
Obese	21 (50.0)	21 (50.0)	42 (100.0)		
**Body fat category**					
Low	83 (78.3)	23 (21.7)	106 (100.0)	12.537	.006*
Normal	103 (68.7)	47 (31.3)	150 (100.0)		
High	64 (59.3)	44 (40.7)	108 (100.0)		
Very High	44 (57.1)	33 (42.9)	77 (100.0)		
**Muscle mass category**					
Low	26 (49.1)	27 (50.9)	53 (100.0)	18.595	.000*
Normal	165 (63.7)	94 (36.3)	259 (100.0)		
High	84 (79.2)	22 (20.8)	106 (100.0)		
Very High	19 (82.6)	4 (17.4)	23 (100.0)		
**Visceral fat category**					
Normal	266 (68.2)	124 (31.8)	390 (100.0)	3.838	.147
High	23 (53.5)	20 (46.5)	43 (100.0)		
Very High	5 (62.5)	3 (37.5)	8 (100.0)		

n= number of participants (frequency).

*Significant at p < 0.01.

**Significant at p < 0.05.

BP = Blood Pressure.

### Determinants of hypertension

A binary logistic regression analysis to investigate factors contributing to the risk of developing hypertension among the PLHIV was conducted. The predictor variables were tested a priori to verify there was no violation of the assumption of multicollinearity (Table 3). The predictor variable, ARV type, in the binary logistic regression analysis was found to contribute to the model. Participants who were on second line ARV had 62% lower odds of developing hypertension (OR = 0.379; 95% CI: 0.169–0.846; p = 0.018) compared with participants who were on first line ARV. Percentage muscle mass was also found to contribute to the model. PLHIV with high percentage muscle mass had 82% lower odds of developing hypertension (OR = 0.177; 95% CI: 0.064–0.487, p < 0.01) compared PLHIV with low percentage muscle mass. Similarly, PLHIV who had very high percentage muscle mass had 78% lower odds of developing hypertension compared to those with low muscle mass (OR = 0.22; 95% CI: 0.051–0.943; p = 0.041).

**Table 3:** Binary logistic regression of hypertension on other factors.

Variable	B	S.E.	Wald	df	Sig.	Exp(B)	95% CI for EXP(B)	
							Lower	Upper
**Type of ART**								
First line ART								Ref.
Second line ART	−0.971	0.41	5.603	1	0.018**	0.379	0.169	0.846
**Does Medication Affect your Appetite?**								
Yes								Ref
No	−0.56	0.386	2.105	1	0.147	0.571	0.268	1.217
**%Body Fat Category**								
Low			2.723	3	0.436			Ref
Normal	0.002	0.419	0	1	0.997	1.002	0.44	2.278
High	−0.408	0.523	0.607	1	0.436	0.665	0.238	1.855
Very High	−0.874	0.661	1.748	1	0.186	0.417	0.114	1.524
**%Muscle Mass**								
Low			11.789	3	0.008			Ref
Normal	−0.741	0.397	3.488	1	0.062	0.476	0.219	1.037
High	−1.734	0.517	11.245	1	0.001*	0.177	0.064	0.487
Very High	−1.516	0.744	4.157	1	0.041**	0.22	0.051	0.943
**BMI Category**								
Underweight			3.953	3	0.267			Ref
Normal	0.546	0.453	1.451	1	0.228	1.725	0.71	4.192
Overweight	0.843	0.547	2.372	1	0.124	2.324	0.795	6.795
Obese	1.398	0.704	3.942	1	0.047**	4.046	1.018	16.083
**IDDS Category**								
Low IDDS			7.13	2	0.028			Ref
Medium IDDS	0.677	0.274	6.098	1	0.014**	1.968	1.15	3.369
Higher IDDS	0.853	0.397	4.614	1	0.032**	2.348	1.078	5.115
Constant	0.705	1.003	0.495	1	0.482	2.025		

*Ref*. = : Reference group.

*Significant at p < 0.01.

**Significant at p < 0.05.

Obesity in the binary logistic regression analysis had 4 times the likelihood of developing hypertension (OR = 4.046, 95% CI: 1.018–16.083; p = 0.047) as compared with underweight**.** Furthermore, PLHIV with medium or high IDDS had approximately twice the odds of developing hypertension compared to those with low IDDS.

Overall, the model is a good one, Chi-square = 68.756, p < 0.01, which accounts for over 20% of the variation in the prevalence of hypertension (*Nagelkerke* pseudo-R square = 0.204).

## Discussion

The study revealed a high prevalence of hypertension among the study participants, a finding consistent with the rising prevalence of non-communicable diseases in SSA [4,25–31]. This rate demonstrates the growing concern of hypertension within this population, particularly as it can complicate HIV disease management and increase morbidity and mortality. While females exhibited slightly higher hypertension prevalence, this difference was not statistically significant, aligning with other studies that found no significant gender disparities in hypertension among PLHIV in similar settings [32–36]. This result is noteworthy as it deviates from the broader literature on hypertension, where men typically present with a higher prevalence, especially in developing countries [37–39]. This discrepancy underscores the unique interplay of factors influencing hypertension in the context of HIV and highlights the need for further investigation into sex-specific risk factors within this population.

Our study identified several key factors associated with hypertension. Notably, individuals on second-line ART were significantly less likely to have hypertension than those on first-line ART. This finding could reflect the metabolic side effects associated with certain first-line ART drugs, as mentioned in the sources, particularly protease inhibitors, which can lead to dyslipidemia, insulin resistance, and fat redistribution, all potentially increasing hypertension risk. However, this finding should be interpreted cautiously. While the study adjusted for several variables, the observed association might be confounded by other unmeasured factors, such as duration of HIV infection or differences in healthcare access and adherence between those on different ART regimens. Further investigation into the specific ART regimens used and their potential link to hypertension is warranted.

The protective effect of higher muscle mass against hypertension, also observed in the study, demonstrates the importance of physical activity and maintaining lean body mass in mitigating hypertension risk among PLHIV. This finding aligns with existing knowledge that physical activity can lower blood pressure and improve cardiovascular health, further emphasizing the need for interventions promoting physical activity in this population [40–42]. Conversely, obesity emerged as a significant risk factor for hypertension, a finding consistent with global trends and highlighting the critical need for weight management strategies within HIV care programs [29,37,39].

Surprisingly, the study found a positive association between dietary diversity and hypertension, contradicting the general understanding that dietary diversity contributes to better overall health and reduced risk of chronic diseases [43]. This finding necessitates further exploration to understand the specific dietary components driving this association. It’s possible that increased consumption of certain food groups, despite greater diversity, may contribute to hypertension risk [44]. For instance, while a more diverse diet might include fruits and vegetables, it could also include higher intakes of processed foods, saturated fats, and sodium, which are known contributors to hypertension. This unexpected finding highlights the importance of examining not just dietary diversity but also the quality and nutrient composition of diets among PLHIV in future research.

Despite its important findings, this study has limitations that should be acknowledged. First, the cross-sectional design precludes establishing causality between the identified factors and hypertension among PLHIV, as exposures and outcomes were measured simultaneously. Second, the use of a hospital-based sample without random selection may introduce selection bias, thereby limiting the generalizability of the findings to the wider population of PLHIV and PLHIV not enrolled in ART care. Third, recall bias and social desirability bias may have affected the accuracy of self-reported lifestyle and dietary information. Additionally, the study was susceptible to potential history or maturation effects, as participants had varying durations on ART and lengths of HIV infection that could influence hypertension risk over time. Furthermore, while the study examined ART regimen type, it did not disaggregate specific drug combinations or cumulative drug exposure. Lastly, the absence of biochemical and inflammatory markers limited the ability to explore underlying biological mechanisms.

Nevertheless, several methodological precautions were taken to reduce the impact of these limitations. To minimize selection bias, participants were recruited from two major ART centers in different settings, capturing a heterogeneous sample representative of typical PLHIV receiving care in Ghana. Standardized and validated instruments were used for blood pressure, anthropometry, and dietary assessment to enhance measurement accuracy and comparability with other studies. The training and supervision of data collectors helped reduce interviewer bias and ensure consistency across sites. Although the study lacked random selection, probability proportional to size (PPS) allocation across facilities improved representativeness within the study area. Furthermore, by restricting inclusion to participants who had been on ART for at least six months, the study partially controlled for maturation effects related to short-term ART initiation and stabilization. Finally, the use of multivariate regression modeling helped to account for potential confounders, thereby improving the robustness and internal validity of the findings.

## Conclusion

This study revealed a high prevalence of hypertension among PLHIV in our study population. Hypertension was significantly associated with obesity and higher dietary diversity, while second-line ART and greater muscle mass appeared to offer protective effects. These findings emphasize the need to integrate hypertension screening and management into routine HIV care to enhance the overall health outcomes of PLHIV. Public health interventions should prioritize the incorporation of regular blood pressure monitoring, nutrition counseling, and weight management strategies within ART programs. Clinicians should be encouraged to assess cardiovascular risk factors as part of comprehensive HIV care. Future research should employ longitudinal and multicenter designs to establish causal relationships and examine the role of specific ART regimens and dietary components in the development of hypertension among PLHIV. Expanding such investigations to include biochemical and inflammatory markers will be critical to elucidating the biological mechanisms underlying these associations.

## Supporting information

S1 File Kasim data hypertension modified. (CSV)
